# Is a Match Better Than No Match? On the Interaction of Demands and Support During Technological Change

**DOI:** 10.3389/fpsyg.2022.824010

**Published:** 2022-06-23

**Authors:** Katharina D. Schlicher, Jannik Reddehase, Günter W. Maier

**Affiliations:** ^1^Division of Work and Organizational Psychology, Department of Psychology, Bielefeld University, Bielefeld, Germany; ^2^Research Institute for Cognition and Robotics (CoR-Lab), Bielefeld University, Bielefeld, Germany

**Keywords:** change demands, change support, social support theory, matching hypothesis, psychological need satisfaction, frustration

## Abstract

Progressing digitalization and technological changes triggered by COVID-19 lockdowns means for organizations that new technologies need to be implemented in shorter time periods. The implementation of new technologies in the workplace poses various change demands on employees. Organizations try to counteract these effects by providing change support in the form of for example training or participation options. However, to date, it is unclear how change demands develop a detrimental effect and whether change support can buffer this relation due to which working mechanisms, and whether the effectiveness of support measures can be increased by matching them to specific change demands. Based on the integrative framework of social support theory, which draws on the job demands-resources model and self-determination theory, we hypothesize that change demands can be most effectively addressed through matching change support. In three consecutive experimental vignette studies (*N*_1_ = 89, *N*_2_ = 134, *N*_3_ = 138) of dependently employed samples, we analyzed the interaction of change demands and change support on attitude to change, satisfaction with the change process, and behavioral intention to use by manipulating the degree of demand (high vs. low) and provided support (high vs. low) and by conducting moderated mediation analyses, and integrated the results meta-analytically. The results show that change demands have a detrimental effect on technology implementation outcomes. In one of the three studies we confirmed a moderating effect of change support. The relation was mediated by perceived frustration, but the mediating effect of psychological need satisfaction was inconclusive. Based on our results, we discuss that the research on matching support requires the evaluation of the personal relevance of the support receiver to increase the chance of achieving a match.

## Introduction

Progressing digitalization and technological change triggered by COVID-19 lockdowns have increased the pace of technological change (Cascio and Montealegre, 2016; Dwivedi et al., 2020). Technological change represents one of many occasions for organizational change (Senior and Swailes, 2010), which is initiated when a new technology or a technology update is introduced at an employee’s workplace (Cascio and Montealegre, 2016). Organizational change in general (e.g., task and role change) can be demanding for employees and can affect employee well-being (Day et al., 2017), their attitudes and attachment to the organization (Sung et al., 2017). Many forms of support are recommended during technological change, and this study examines how to most effectively design change support as a means of addressing change demands.

The implementation of new technologies in the workplace temporarily disrupts work and often proves demanding to employees, even if the implemented technology is well-developed (Demerouti, 2020). Change demands can result from work design changes that can occur when employees, for example, have to take up new tasks for which they do not have the required skills, when their level of job autonomy shifts, when their working routines change (Demerouti, 2020; Parker and Grote, 2020), or because the implemented technology requires intensive customization (Momoh et al., 2010). Overwhelming change demands can result in resistance to change in employees (Oreg et al., 2018), expressed for example in the refusal to use the new technology.

Organizations intend to help their employees to face these change demands by providing change support, for example by offering training, options to participate in the change process, technical support, or additional resources (Oreg et al., 2011; Iden and Eikebrokk, 2013; Reitsma and Hilletofth, 2018). These support strategies have been meta-analytically shown to directly affect technology implementation success with a medium-sized positive effect (Schlicher and Maier, 2019a). Yet, whether change support also interact with demands and buffer their detrimental effect when designed accordingly, and if this interaction could potentially increase the effect of change support interventions, is less well-researched. This knowledge is of high practical value as it could be used to design more effective change management support measures.

From the theoretical perspective, we are going to elaborate on one of the most central assumptions of social support theory, the matching principle. Jolly et al. (2021) published a comprehensive review article on social support theory criticizing that its model assumptions on support-demand matches lack systematic research and empirical confirmation. Previous research did not yield conclusive results on the moderating role of support (e.g., Viswesvaran et al., 1999; Mathieu et al., 2019), but it was mostly conducted as field research in which support was not specifically designed to match a particular demand. In the context of technological change, providing support is recommended, yet previous research has not systematically analyzed whether particular support interventions were designed as a means to counteract demands of technological change (Schlicher and Maier, 2019a), or whether an interaction could increase the effectivity of support interventions.

Therefore, we pursue the research question of how change demands have a detrimental effect on technology implementation outcomes, and whether change support can buffer this effect, especially when it is designed according to the matching principle of social support theory. Furthermore, we analyze which mechanisms mediate the relation between change demands and change support on technology implementation outcomes, because this knowledge also helps to design more effective measures to support change.

To understand demands during technological change in particular and the counteracting effect of change support, we applied the integrative framework introduced by Jolly et al. (2021) which relates to Job Demands-Resources Theory (JD-R; Bakker and Demerouti, 2007) and Self-Determination Theory (SDT; Deci and Ryan, 2000; Deci et al., 2017) to explain the joint effect of social support and demands on work outcomes with intrapersonal processes. We applied an experimental research approach, as proposed by Jolly et al. (2021) to allow for causal inferences to be drawn in a controlled research environment, and conducted three consecutive vignette studies. Although experimental vignette methodology is often criticized for its low external validity due to its hypothetical nature (Aguinis and Bradley, 2014), it has the advantage of testing research questions that are not readily observable in practice and manipulating antecedent conditions to infer causal relationships in a parsimonious manner.

We contribute to the literature by first increasing the understanding of the nature of change demands. As of today, there has been less research on change demands as compared to change support (e.g., Smollan, 2015). Second, we elaborate how change support interacts with change demands, and provide clarity on this key model assumption of social support theory. In practice, many change support interventions might be too general to have an effect on a specific change demand (e.g., the provision of management support for the general implementation of the new technology when handling the new technology requires specific training). Third, we analyze the mechanisms by which change demands develop detrimental effects and how change support can buffer this relation in order to be able to design more efficient change support interventions. Fourth, by applying an experimental research design, we were able to systematically manipulate and observe the consequences of the interaction of change demands and change support, thereby being able to analyze the causal relationship of the two. In doing so, we further enhance the understanding of change management processes.

## Interaction of Change Demands and Change Support

We followed the above outlined research questions by first describing change demands that can occur during technological change and analyzing the effects of change demands on technology implementation outcomes (see section “Shifting the Research Focus to Change Demands”), then by explaining support according to social support theory and paths of interaction between demands and support on these outcomes (see section “Easing the Effect of Change Demands by Providing Change Support”), and third by analyzing the mechanisms of these relations (see section “Explaining the Mechanisms of Change Demands”).

### Shifting the Research Focus to Change Demands

The nature of demands is explained by the JD-R (Bakker and Demerouti, 2007), which states that job demands (defined as facets of the job that require physical or psychological effort and are costly to the individual) are ever-present in every workplace and negatively affect well-being and performance through perceived strain. As a variant of job demands, change demands represent specific job demands that occur during the change process.

Various demands can occur during technological change. Although the result of implementing a new technology should be beneficial to the employee (e.g., process optimization, reduction in system disruptions) and free up resources, the process of making the transition can be demanding. Change demands can result from the requirement to adapt to the alteration of work processes (Momoh et al., 2010; Ali et al., 2016; Demerouti, 2020) or a temporarily increased workload during implementation (Smollan, 2015; Carlson et al., 2017). Change demands can also result from the alteration of job contents (Ali et al., 2016). Examples of job content change are the introduction of tasks or technology features that require the employee to develop new skills to perform the task and handle the technology (Oberländer et al., 2020; Paruzel et al., 2020), or the uptake of new work roles that the employee did not have to play beforehand (Smollan, 2015). Also, work design characteristics such as the perceived level of autonomy of a job might alter (Parker and Grote, 2020). Furthermore, change demands can result from the technology itself, for example when the technology lacks quality, employees perceive a lack of control over the technology, or the technology allows for less effective communication but increased external monitoring (Momoh et al., 2010; Day et al., 2012).

In addition to the above descriptive differentiation of demands, change demands can also be differentiated dimensionally, and this knowledge may be used for optional support matching. With reference to social support theory, Cutrona (1990) clustered stressful events to describe optimal support matching. One dimension is the controllability of the situation, which technological change offers at both extremes. Some demands can be controlled by employees, such as learning the new technology functions, and require instrumental support. Other demands feel uncontrollable to workers, e.g., job design and work role changes, and are best managed with emotional support. Thereby, change demands can be rather instrumental (i.e., physical) in nature, especially when the technology implementation leads to tasks that require new skills in order to successfully perform them; alternatively, change demands can be emotional (i.e., affective) in nature, when employees feel insecure while up taking new work roles or having to work with new colleagues (e.g., House, 1981; de Jonge et al., 2008).

Another aspect of why change demand may have a differential effect on technology implementation outcomes could be the amplitude to which they occur. Depending on the scope of technological change (e.g., Street and Gallupe, 2009), that is, how extensive the task and role changes are as a result of the technology introduced and how many different demands are placed on employees, change can occur at different amplitudes. Ample technology implementation projects that largely affect how work is conducted and how work teams interact can lead to diffuse change demands for employees (e.g., new tasks and roles, increased workload, adapting to the new technology, letting go of old work routines, working with new colleagues, technical problems with the new technology; Smollan, 2015). More closely circumcised technology implementation projects can lead to specific change demands, for example the alteration of work tasks might lead to new skill requirements or the alteration of new work roles (Smollan, 2015; Paruzel et al., 2020).

Change demands can lead to the failure of technology implementation processes. The failure or success of a technology implementation process can be measured in change-specific outcomes such as attitude to change (Oreg, 2006), satisfaction with the implementation process, and behavioral intention to use the new technology (Davis et al., 1989). Attitude to change describes an employee’s mindset about the change process in three dimensions: affective (e.g., strain or joy experienced during the change), cognitive (e.g., evaluation of the change’s risks or chances), and behavioral attitude to change (e.g., intention to hinder or promote the change) (Oreg, 2006). Positive attitude to change is often viewed as a prerequisite of a multitude of other change supportive behaviors (e.g., Rafferty et al., 2013), but can also lead to counterproductive change behaviors when negatively affected by high change demands (Oreg et al., 2018). Satisfaction with the change process represents a concretization of the general construct job satisfaction (Cammann et al., 1983). When high demands occur during the change process, the satisfaction with the conduction of the change process decreases. Behavioral intention to use is a construct of the technology acceptance model that describes whether employees are motivated to use the implemented technology (Davis et al., 1989; Venkatesh and Bala, 2008). Behavioral intention to use is predicted by an evaluation of the technology (perceived ease of use, usefulness), but also by external variables such as facilitating conditions of the change context (Venkatesh et al., 2012). When the technology itself or the facilitating conditions of the change process are perceived as low in quality, behavioral intention to use the technology decreases (e.g., Rajan and Baral, 2015). Behavioral intention to use must be distinguished from actual usage. While actual usage measures how often the new technology is used and can easily be affected, for example, by the organization’s commitment to use the new technology, behavioral intention to use measures the motivation to use the new technology. Although the use of a new technology may be mandatory, internal willingness to use depends on aspects of the technology and the implementation process. Yet, research on the effects of change demands is sparse (e.g., Smollan, 2015) and has not been systematically investigated in the context of technological change. We assume that change demands in general will negatively affect the three technology implementation outcomes.

*Hypothesis 1:* Change demands negatively affect (a) positive attitude to change, (b) satisfaction with the change process, and (c) behavioral intention to use.

### Easing the Effect of Change Demands by Providing Change Support

Organizations are often aware that technological change can prove challenging to employees and therefore provide support (Iden and Eikebrokk, 2013; Reitsma and Hilletofth, 2018). Applying Jolly et al.’s (2021) definition of social support, change support takes the *form* of behaviors performed by members of the organization as the *source* of support and is measured through the perception of support received by employees. JD-R groups change support as a job resource (Demerouti, 2020) that is defined as facet of the job that reduces job demands, stimulates growth, and helps achieve work goals (Bakker and Demerouti, 2007).

In accordance with change demands, change support can take varying *forms*, too. During technological change, organizations have a multitude of possible support interventions at their disposal: training, technical support, information on the upcoming change, support from management, and options to participate, among others (for an overview: Oreg et al., 2011; Iden and Eikebrokk, 2013; Reitsma and Hilletofth, 2018; Schlicher and Maier, 2019a). Social support theory (House, 1981) states that four kinds of support can be distinguished. Instrumental support represents the provision of resources required to solve a problem (e.g., money), informational support represents the provision of knowledge to help oneself (e.g., information on an open job position), emotional support represents the provision of care and sympathy, and appraisal support represents the provision of feedback from others (Smollan, 2017). Meta-analysis showed that workplace and change support is effective in increasing positive and lowering negative attitudes and behaviors (Mathieu et al., 2019; Schlicher and Maier, 2019a).

Job Demands-Resources Theory states that demands and resources not only directly impact work and change-specific outcomes, but also interact to affect outcomes. The relation of support with demands is that it positively affects outcomes (such as strain) directly, but also buffers the relation of demands (such as stress) and these outcomes (Viswesvaran et al., 1999). Field research that examined the interaction between change support (management support, training, and participation) and change demands during technological change found no interaction effect, but the reason may be that demands were considered low and that organizations have not paid enough attention to matching support with specific demands (Schlicher and Maier, 2019b). Therefore, we choose an experimental research setting, where the matching design of change support and change demands is controlled, and assume that the effect of change demands will be moderated by change support.

*Hypothesis 2:* Change support moderates the negative relationship of change demands and (a) attitude to change, (b) behavioral intention to use the system, and (c) satisfaction with the change process.

### Explaining the Mechanisms of Change Demands

Change demands have a detrimental effect on outcomes of change (e.g., Oreg et al., 2011, 2018). However, it is not yet clear which mechanisms underlie this relation. A theory that allows for the derivation of assumptions about how change demands and change support jointly affect outcomes by describing intrapersonal processes is SDT (Deci and Ryan, 2000; Deci et al., 2017). The integration of social support theory, JD-R, and SDT further fosters a deeper understanding of the social support processes of work (Jolly et al., 2021) to develop support according to intrapersonal effects of demands on employees.

The SDT is a motivational theory proposing that three basic psychological needs must be fulfilled in order for motivation, performance, and well-being to occur. In the context of work, this means that employees must feel competent to have their need for competence fulfilled, employees must feel part of a group to have their need for relatedness fulfilled, and employees need to perceive having freedom of choice to have their need for autonomy fulfilled (Deci and Ryan, 2000; Deci et al., 2017). High change demands can lead to a frustration of psychological needs (van den Broeck et al., 2016) and stress (Olafsen et al., 2017). For example, changes in work tasks and work ambiguities were associated with burnout via need thwarting (Gillet et al., 2015), workplace bullying led to burnout and lowered engagement via need dissatisfaction (Trépanier et al., 2013), and job insecurity was associated with emotional exhaustion via need frustration (Vander Elst et al., 2012). During technological change, a change in work tasks and skill requirements can lead to dissatisfaction of the need for competence, work design changes can lead to dissatisfaction of the need for autonomy, and when employees have to work together in new work groups, they can perceive that their need for relatedness is dissatisfied (Schlicher and Maier, 2019b). The provision of support intends to accompany change in a way that fosters the experience of need fulfillment at work (van den Broeck et al., 2016). For example, organizational resources such as perceived justice were found to reduce need thwarting (Gillet et al., 2015), and fostering an understanding of the rationale of the change, feeling acknowledged, and having a choice during change can lead to acceptance of change (Gagné et al., 2000). SDT does not explicitly predict an interaction of demands and support on need fulfillment, and Jolly et al. (2021) integrated JD-R and SDT on the motivational path. However, as van den Broeck et al. (2016) noted, research on need dissatisfaction is underrepresented and requires integration with related theories. We therefore propose that experiences of need dissatisfaction explain the experiences of strain and frustration following the occurrence of demands. As predicted in JD-R, support should buffer the effect of demands on strain, which is mediated by need dissatisfaction. In line, psychological need satisfaction (and subsequently its dissatisfaction) is associated with performance, satisfaction, and commitment (van den Broeck et al., 2016).

The lack of satisfaction of the three psychological needs can explain observations of frustration during technological change (e.g., Castillo et al., 2018; Gray et al., 2020). Frustration refers to affective reactions to inhibiting work conditions (Peters et al., 1980). Trépanier et al. (2015) found that need frustration following the occurrence of job demands led to psychological distress in employees, but the provision of job resources decreased need frustration. The affective state of frustration following the lack of need satisfaction might explain why technology implementation outcomes are negatively affected.

*Hypothesis 3:* The relationship of change demands and (a) attitude to change, (b) satisfaction with the change process, and (c) behavioral intention to use will be serially mediated by need satisfaction and frustration. Change support moderates the mediation process.

**FIGURE 1:**
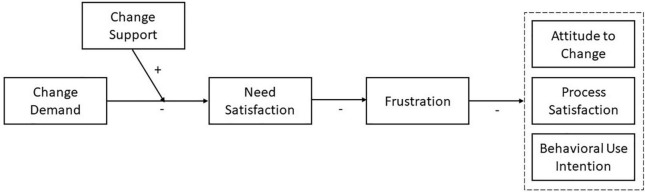
Model assumption.

Model assumptions are summarized in Figure 1.

## Materials and Methods of Study 1

### General Methodological Approach to the Three Studies

To test the model’s assumptions, we applied an experimental between-subjects vignette study design in three consecutive studies and integrated their results meta-analytically. We chose this approach because it allowed us to systematically test the assumptions of the model in a parsimonious design and controlled environment, to derive causal conclusions, and because the subject of the study is not easily observed in practice, as change managers are not yet advised to follow the matching principle when designing interventions to support change. We maintained this approach in all three studies, because the replication crisis in psychology has highlighted the importance of conducting several studies in comparable research settings to reach conclusions on true effects (Maxwell et al., 2015; Shrout and Rodgers, 2018). We applied paper people vignettes, in which study participants are typically presented with written scenario texts of a hypothetical situation and asked to indicate their attitudes, affects and behavioral intentions. This type of vignettes is appropriate for the assessment of explicit processes and responses that study participants can reflect about (Aguinis and Bradley, 2014), as is the case with responses to change. This type of experimental manipulation is widely used in research concerned with an in-depth analysis of a working mechanism in question, as was the purpose of this study (e.g., Keck and Babcock, 2018; Abraham et al., 2019). The experimental vignette methodology increases internal validity by systematically manipulating the independent variables of change demands and change support, but has been criticized for its lower external validity. We increased the external validity of the vignettes by describing the scenario as realistically as possible for study participants, providing contextual information for immersion, and relying on reports of technological changes in practice to describe task changes. We also increased external validity by recruiting study participants who could more easily empathize with the situation described because they were dependently employed and had personal experience with change (Aguinis and Bradley, 2014). The length of the vignette text was similar in each of the studies to exclude method effects. In each study, we manipulated the degree of demand experienced (high vs. low) and support provided (high vs. low) in three scenarios (we will explain the manipulations for each study in its respective section). Study participants were randomly assigned to the experimental groups. The studies were conducted online on the platform Qualtrics. The ethical committee of the university approved the study designs prior to conduction (Study 1 #2020-058, and Studies 2 and 3 #2020-167).

**FIGURE 2:**
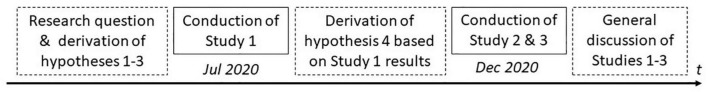
Timeline of Studies 1–3 conduction.

We will present the three studies in the order in which they were conducted. The approach is summarized in Figure 2. First, we conducted Study 1, in which we analyzed the interaction of high-amplitude change demands with different types of change support. After interpreting the results of Study 1, we derived another hypothesis based on a theoretical extension of social support theory and refined the design of Study 2 and 3 by testing a single change demand with corresponding change support in each case. Accordingly, we will present Study 1 in its entirety before presenting Studies 2 and 3. In Study 1, we analyzed whether the mixed effects of technology implementation (workplace changes to tasks, routines, and social interactions) can be buffered by mixed acts of support (training, participation options, information, and management support).

### Research Sample of Study 1

Prior to participant recruitment, we calculated the required sample size with G*Power (Faul et al., 2007). For the first study, the estimation of the required sample size was based on the parameters of a field study that recruited participants with similar demographics on a related topic (Schlicher and Maier, 2019b). The *a priori F*-test (ANOVA, main effects and interactions) results showed that between *N* = 34 (attitude to change, *r*_mean_ = 0.45), *N* = 75 (process satisfaction, *r*_mean_ = 0.31) and *N* = 971 (behavioral use intention, *r*_mean_ = 0.09) participants needed to be recruited in order to detect an effect of demand or support on the outcomes.

Study participants were recruited via social media in 2020 in Germany and were offered to take part in a prize draw of a 4 × 10 € shopping voucher. In total, *N* = 168 answered the call. To be included into the analysis, participants needed to give their approval for anonymous data storage and analysis, the survey needed to be filled out completely, and at least two out of three attention checks needed to be answered correctly. Participants needed to work at least 20 h per week in order to be assumed to know work processes in organizations well and be able to fully immerse in the description of the fictitious change situation of the study’s manipulation to increase external validity. They had to be dependently employed, for we expected that self-employed and corporate management would be in charge of designing change processes and therefore answer research questions from a different perspective than intended (see also: discrepancy of evaluations between provided vs. received support; Jolly et al., 2021). In order to detect low attention while reading research items, we excluded participants who were three times quicker (3 *SD*s) than the median of total processing duration and time reading the vignette text (Leiner, 2019). After applying the inclusion criteria, *N* = 89 participants were included in the final sample.

Study participants were on average 34.07 years old (*SD* = 11.65; range: 20–59 years), 33.3% male. On average, they worked 35.32 h per week (*SD* = 7.21; range: 18–45 h), in a dependently employed (85.7%), marginally employed (1.2%), or part-time employment position alongside studies or training (13.1%). The majority of participants had a university degree (39.2%) or vocational training (22.6%). Most participants held no leadership responsibilities (70.2%), while 25% had leadership responsibilities for teams or projects, and 4.8% on some higher hierarchy level. Most participants worked in health services (26.2%), commercial services (15.7%), or administrative services (14.5%). Additionally, 81% of the participants had experienced technology implementation in their workplace; on average, they judged the implementation as slightly good (6.24, *SD* = 2.24; range 1–10). Study participants evaluated themselves as average tech-savvy (5.15, *SD* = 2.67, range 0–10).

### Procedure and Manipulation of Study 1

First, study participants were informed of the study’s outline and their data privacy rights. Second, the correctness of filtering factors (required minimum working hours of 20 h per week) had to be confirmed. Third, vignette texts and questionnaires were presented, followed by demographic information. Lastly, information concerning compensation for participation was provided.

For the means of manipulation, participants were asked to read a scenario text concerning the implementation of a new technology in the workplace, and subsequently answer questions how they would react if they had experienced this change. The scenario text began with an explanation of the context of the hypothetical situation. This approach was the same for all studies and manipulations and included the name of the company, an explanation of the work tasks that are being changed, and the progress of technology implementation. Participants were asked to imagine they worked as an accountant for the fictitious company portrayed in the text. The participants were told that the fictitious company is in the process of changing a number of aspects in their imagined workplace due to the implementation of a new computer system. The study manipulation was then twofold. First, participants were informed whether the technology implementation would lead to more or less ample changes to their daily work. The scenario described that tasks and routines would change due to the technology implementation. In the high demands condition, participants were informed that the technology will require them to do their tasks differently and how much daily work routines will change. In the low demands condition, participants were informed that many tasks will remain unchanged by the introduction of the technology and that there will be little change in work routines. Second, participants were informed whether or not they received a form of organizational support to counteract the change demands. In the high support condition, participants could benefit from different support interventions (training, options to participate, support from management, and information on the change). In the low support condition, participants were informed that they have not received these support options, because no budget was allocated or management did not have enough time to address the concerns. Vignette texts and a table indicating the respective manipulations are available for inspection in the Supplementary Material File.

### Measures

Items were presented in German on a seven-point Likert-scale, if not stated otherwise. Scales for which there was no German translation available were translated following collaborative and iterative translations guidelines (Douglas and Craig, 2007). Items per scale as well as mediator and outcome variables per page were presented in randomized order.

We tested for the successful manipulation of change demands by applying three items of the individual job impact scale (exemplary statement: “The work processes and procedures I use have changed,” on a five-point Likert scale; Caldwell et al., 2004). The successful manipulation of change support was secured by assessing whether study participants recognized the availability of support (e.g., “Overall, I feel appropriately involved in the rollout of the new program.”) and whether the presentation of support options led to the perception of perceived organizational support at work (three items, e.g., “My organization cares about my opinions”; Eisenberger et al., 1997).

Two mediator variables were assessed: psychological need satisfaction, and perceived frustration associated with the change. Need satisfaction was assessed with 18 items of a German translation (Martinek, 2014) of the Work-related Basic Need Satisfaction scale (e.g., “I really master my tasks at my job” on a five-point Likert scale; van den Broeck et al., 2010). Frustration was assessed with three items of the organizational frustration scale (Peters et al., 1980) that was adapted to measure frustration in the workplace (Gray et al., 2020). An exemplary item is “Doing this work during the change is a very frustrating experience.” Study participants were instructed to immerse themselves again in the work situation described in the vignettes and respond how they would react in that situation before each of the need satisfaction, frustration, and outcome scales were presented.

Three outcome variables were assessed: attitude to change, satisfaction with the change process (in the following called process satisfaction), and behavioral intention to use the new technology. Attitude to change was assessed with 15 items by Oreg (2006). Item formulation was changed to the present tense (e.g., “I think that it’s a negative thing that we are going through this change.”). Process satisfaction was assessed with three items of the Michigan Organizational Assessment Questionnaire Job Satisfaction Subscale (Cammann et al., 1983) that were linguistically formulated to fit the change process (e.g., “All in all I am satisfied with the technology implementation process.”). Behavioral intention to use was assessed with three German five-point Likert scale items (Kohnke and Müller, 2010) based on assumptions of the technology acceptance model (Davis et al., 1989) and on item formulations by Taylor and Todd (1995) and Amoako-Gyampah and Salam (2004) (e.g., “I am motivated to use the new technology”).

### Data Analysis

Data analysis was conducted in SPSS version 27. For the coding of the manipulation variables, 0 was chosen for low demand and low support groups, and 1 was chosen for high demand and high support groups. *T*-tests were calculated to estimate the mean difference between the high and low manipulation groups. For hypothesis testing, we followed recommendations by Hayes (2018), applying Model 1 for moderation analyses and Model 85 for conditional model analyses. Finally, we integrated the results of the three studies meta-analytically, following recommendations of Cheung (2015). Meta-analysis was performed in R using the metaSEM package (see Cheung, 2015).

## Results and Discussion of Study 1

### Manipulation Check and Descriptive Statistics

The manipulation of change demands was successful [*t*(82) = 9.51, *p* < 0.001, *d* = 0.93]. The manipulation of change support was also successful. The mean difference for availability of support [*t*(82) = 20.80, *p* < 0.001, *d* = 0.66] as well as perceived organizational support was significant [*t*(82) = 17.71, *p* < 0.001, *d* = 1.00].

The mean differences and standard deviations of study variables of the three studies are portrayed in Table 1. Overall, mean differences per manipulation show in the intended direction. Table 2 shows the correlations of study variables. As expected, change demands affected study variables negatively whereas change support affected study variables positively. The reliability of the measures is also portrayed in Table 2.

**TABLE 1:** Means and standard deviations of study variables.

			Need satisfaction	Frustration	Attitude to change	Process satisfaction	Behavioral use intention
			*M* (*SD*)				
Study 1	High demand	*N* = 44	3.26 (0.68)	4.35 (1.49)	4.55 (1.04)	4.13 (1.54)	3.84 (0.85)
	Low demand	*N* = 40	3.40 (0.60)	3.23 (1.46)	5.27 (1.04)	4.76 (1.55)	4.12 (0.78)
	Cohen’s *d*		0.65	1.47	1.04	1.55	0.82
	High support	*N* = 43	3.62 (0.51)	3.12 (1.26)	5.45 (0.87)	5.38 (1.19)	4.14 (0.73)
	Low support	*N* = 41	3.02 (0.63)	4.55 (1.54)	4.31 (1.01)	3.43 (1.27)	3.80 (0.89)
	Cohen’s *d*		0.57	1.41	0.94	1.23	0.81
Study 2	High demand	*N* = 68	3.13 (0.64)	4.70 (1.31)	4.48 (1.19)	4.10 (1.68)	3.97 (0.83)
	Low demand	*N* = 66	3.59 (0.43)	2.75 (1.24)	5.63 (0.77)	5.34 (1.04)	4.33 (0.61)
	Cohen’s *d*		0.55	1.27	1.01	1.40	0.73
	High support	*N* = 69	3.47 (0.47)	3.58 (1.49)	5.32 (0.94)	5.21 (1.12)	4.28 (0.70)
	Low support	*N* = 65	3.23 (0.68)	3.91 (1.71)	4.75 (1.29)	4.18 (1.72)	4.02 (0.79)
	Cohen’s *d*		0.58	1.60	1.13	1.44	0.74
Study 3	High demand	*N* = 69	3.23 (0.53)	4.31 (1.14)	4.64 (0.97)	4.42 (1.41)	4.15 (0.75)
	Low demand	*N* = 69	3.49 (0.50)	2.96 (1.13)	5.26 (1.02)	4.99 (1.33)	4.15 (0.70)
	Cohen’s d		0.51	1.13	0.99	1.37	0.72
	High support	*N* = 69	3.51 (0.49)	3.34 (1.26)	5.29 (0.88)	5.41 (1.01)	4.36 (0.65)
	Low support	*N* = 69	3.21 (0.53)	3.93 (1.31)	4.61 (1.08)	4.00 (1.38)	3.93 (0.72)
	Cohen’s *d*		0.51	1.29	0.98	1.21	0.69

*Studies 1–3, N per experimental manipulation condition.*

**TABLE 2:** Correlation matrix of Study 1.

	1	2	3	4	5	6	7
(1) Demand (0 = low, 1 = high)	0.84						
(2) Support (0 = low, 1 = high)	–0.02	0.96/0.87					
(3) Need satisfaction	–0.08	0.38**	0.87				
(4) Frustration	0.32**	–0.42**	–0.48**	0.66			
(5) Attitude to change	–0.24**	0.43**	0.65**	–0.65**	0.90		
(6) Process satisfaction	–0.19	0.58**	0.59**	–0.58**	0.78**	0.76	
(7) Behavioral use intention	–0.13	0.20*	0.49**	–0.36**	0.57**	–0.55**	0.84

*N = 84. Reliability estimates in Cronbach’s alpha on the diagonal.*

*p < 0.05, **p < 0.01.

### Interaction of Change Demands and Change Supports

The results of the analyses of Hypotheses 1 and 2 are portrayed in Table 3. In Study 1, mixed demands of task and role changes following technology implementation were investigated. The results show that mixed change demands significantly lowered attitude to change (*b* = –0.75, *p* = 0.01), but not process satisfaction (*b* = –0.41, *p* = 0.29) or behavioral intention to use (*b* = –0.18, *p* = 0.47). Mixed change support, manipulated as giving four different kinds of support following (House, 1981), did not significantly moderate the relation of change demands and outcomes, yet change support significantly improved attitude to change (*b* = 1.07, *p* < 0.001) and process satisfaction (*b* = 2.11, *p* < 0.001). Therefore, for Study 1, Hypothesis 1 can only be supported for attitude to change, and Hypothesis 2 cannot be supported.

**TABLE 3:** Moderation analysis of Studies 1–3.

		(1) DV: Attitude to change			(2) DV: Process satisfaction			(3) DV: Behavioral use intention		
	Predictor	*b* (*SE*)	*t*	*p*	*b* (*SE*)	*t*	*p*	*b* (*SE*)	*t*	*p*
Study 1	Constant	4.71 (0.20)	23.26	<0.001	3.65 (0.28)	13.18	<0.001	3.90 (0.19)	20.98	<0.001
	Demand	–0.75 (0.28)	–2.72	0.01	–0.41 (0.38)	–1.08	0.29	–0.18 (0.25)	–0.72	0.47
	Support	1.07 (0.28)	3.83	<0.001	2.11 (0.38)	5.53	<0.001	0.43 (0.26)	1.65	0.10
	Demand x Support	0.12 (0.39)	0.30	0.77	–0.34 (0.53)	–0.65	0.62	–0.17 (0.35)	–0.47	0.64
		*R*^2^ = 0.38, *MSE* = 0.78 *F*(3, 80) = 16.14, *p* < 0.001			*R*^2^ = 0.43, *MSE* = 1.46 *F*(3, 80) = 20.01, *p* < 0.001			*R*^2^ = 0.07, *MSE* = 0.66 *F*(3, 80) = 2.09, *p* = 0.11		

Study 2	Constant	5.55 (0.16)	33.87	<0.001	5.09 (0.22)	23.00	<0.001	4.27 (0.13)	34.03	<0.001
	Demand	–1.61 (0.23)	–6.91	<0.001	–1.85 (0.32)	–5.87	<0.001	–0.52 (0.18)	–2.92	0.00
	Support	0.17 (0.23)	0.72	0.47	0.50 (0.31)	1.58	0.12	0.12 (0.18)	0.68	0.50
	Demand x Support	0.86 (0.33)	2.63	0.01	1.14 (0.44)	2.58	0.01	0.30 (0.25)	1.18	0.24
		*R*^2^ = 0.35, *MSE* = 0.89 *F*(3,130) = 23.66, *p*.001			*R*^2^ = 0.32, *MSE* = 1.62 *F*(3,130) = 20.66, *p* < 0.001			*R*^2^ = 0.10, *MSE* = 0.52 *F*(3,130) = 4.86, *p* = 0.00		

Study 3	Constant	4.85 (0.16)	30.68	<0.001	4.25 (0.20)	21.39	<0.001	3.82 (0.12)	33.98	<0.001
	Demand	–0.50 (0.23)	–2.24	0.03	–0.51 (0.28)	–1.81	0.07	0.23 (0.17)	1.39	0.17
	Support	0.82 (0.23)	3.64	0.00	1.51 (0.28)	5.33	<0.001	0.66 (0.17)	4.01	<0.001
	Demand x Support	–0.25 (0.32)	–0.79	0.43	–0.17 (0.40)	–0.42	0.68	–0.46 (0.23)	–1.98	0.049
		*R*^2^ = 0.21, *MSE* = 0.88 *F*(3,134) = 11.61, *p* < 0.001			*R*^2^ = 0.31, *MSE* = 1.38 *F*(3,134) = 19.70, *p* < 0.001			*R*^2^ = 0.12, *MSE* = 0.47 *F*(3,134) = 5.84, *p* < 0.001		

*Study 1: N = 84; manipulation: mixed demands of task and role changes, mixed support of four kinds following House (1981). Study 2: N = 134; manipulation: work task changes as demand, training as support. Study 3: N = 138; manipulation: work role changes as demand, participation as support.*

### Mechanisms of the Effect of Change Demands

In Hypothesis 3, we assumed that the effect of change demands on outcomes was mediated through a serial mediation of psychological need satisfaction and frustration that is moderated through change support. In Study 1 (Table 4), change demands did not significantly negatively affect need satisfaction (*b* = –0.18, *p* = 0.31), yet need satisfaction significantly affected frustration (*b* = –0.72, *p* = 0.00) and frustration significantly affected attitude to change (*b* = –0.22, *p* < 0.011) and process satisfaction (*b* = –0.25, *p* = 0.01), but not behavioral intention to use (*b* = –0.12, *p* = 0.06). Change support did not moderate any of the relations, yet was significantly positively related with need satisfaction (*b* = 0.54, *p* = 0.00) and process satisfaction (*b* = 1.44, *p* < 0.001). The direct effect was not significant for each of the outcomes, nor was the mediation via need satisfaction. Yet, the indirect effect of mediation by frustration was significant for attitude to change (95% CI = [–0.54; –0.05] and [–0.41; –0.02]) and process satisfaction (95% CI = [–0.63; –0.04] and [–0.47; –0.01]). Therefore, Hypothesis 3 cannot be supported for Study 1.

**TABLE 4:** Regression coefficients, standard errors, and model summary information of the moderated mediation model – Study 1.

	Consequent														
	(M1) Need satisfaction			(M2) Frustration			(Y1) Attitude to change			(Y2) Process satisfaction			(Y3) Behavioral use intention		
Antecedent	*b (SE)*	*t*	*p*	*b (SE)*	*t*	*p*	*b (SE)*	*t*	*p*	*b (SE)*	*t*	*p*	*b (SE)*	*t*	*p*
Constant	3.12 (0.13)	23.58	<0.001	6.09 (0.81)	7.56	<0.001	3.43 (0.59)	5.81	<0.001	2.33 (0.90)	2.59	0.01	2.63 (0.61)	4.35	<0.001
(X) Demand	–0.18 (0.18)	–1.01	0.31	1.18 (0.39)	3.00	0.00	–0.34 (0.23)	–1.47	0.15	0.05 (0.35)	0.14	0.89	0.08 (0.24)	0.33	0.74
(W) Support	0.54 (0.18)	2.94	0.00	–0.77 (0.42)	–1.86	0.07	0.45 (0.24)	1.90	0.06	1.44 (0.36)	3.99	<0.001	–0.02 (0.24)	–0.06	0.95
Demand x Support	0.11 (0.25)	0.44	0.66	–0.37 (0.55)	–0.67	0.50	–0.06 (0.31)	–0.19	0.85	–0.53 (0.46)	–1.14	0.26	–0.28 (0.31)	–0.90	0.37
(M1) Need Satisfaction	–	–	–	–0.72 (0.24)	–3.00	0.00	0.68 (0.14)	4.77	<0.001	0.73 (0.21)	3.37	0.00	0.56 (0.15)	3.81	<0.001
(M2) Frustration	–	–	–	–	–	–	–0.22 (0.06)	–3.49	< 0.001	–0.25 (0.10)	–2.59	0.01	–0.12 (0.06)	–1.89	0.06
	*R*^2^ = 0.23		*R*^2^ = 0.40			*R*^2^ = 0.62			*R*^2^ = 0.57			*R*^2^ = 0.30			
	*F*(3,80) = 7.90, *p* < 0.001		*F*(4,79) = 13.16, *p* < 0.001			*F*(5,78) = 25.72, *p* < 0.001			*F*(5,78) = 20.92, *p* < 0.001			*F*(5,78) = 6.65, *p* < 0.001			
Direct effect (95% CI)	W = 0						[–0.80; 0.12]			[–0.65; 0.75]			[–0.39; 0.55]		
	W = 1						[–0.83; 0.04]			[–0.1.14; 0.18]			[–0.65; 0.24]		
Ind. effect M1 (95% CI)	W = 0						[–0.41; 0.15]			[–0.49; 0.15]			[–0.34; 0.12]		
	W = 1						[–0.29; 0.17]			[–0.33; 0.19]			[–0.23; 0.14]		
Ind. effect M2 (95% CI)	W = 0						[–0.54; –0.05]			[–0.63; –0.04]			[–0.35; 0.02]		
	W = 1						[–0.41; –0.02]			[–0.47; –0.01]			[–0.29; 0.01]		
Ind. effect M1 + M2 (95% CI)	W = 0						[–0.13; 0.03]			[–0.15; 0.03]			[–0.09; 0.02]		
	W = 1						[–0.07; 0.04]			[–0.08; 0.05]			[–0.05; 0.03]		

*N = 84. Manipulation: mixed demands of task and role changes, mixed support of four kinds following House (1981).*

### Discussion of Study 1

Change demands negatively affect attitude to change, but not satisfaction with the change process or the behavioral intention to use the technology. Change support does not moderate the effect of change demands. Yet, change support has a significant direct effect on attitude to change and satisfaction with the change process. The relation was mediated through perceived frustration, but not need satisfaction. The general provision of change support as mixed change intervention might have been too unspecific to affect the described high amplitude, mixed demands in the vignette scenario.

A theoretical extension of the social support theory has not been considered so far, the matching hypothesis of social support theory. We will further elaborate whether a stronger match of specific change demands and change support following principles of social support theory (House, 1981; Cohen and Wills, 1985), focusing on specific demands and matching support, will lead to an interaction and in effect to stronger effects of the provision of change support interventions, and test its assumptions in Studies 2 and 3.

## Match of Change Demands and Change Support

Social support theory (House, 1981), especially its matching hypothesis (Viswesvaran et al., 1999) and the triple match principle (de Jonge and Dormann, 2006), concretize the assumptions of JD-R by describing under which conditions an interaction of change demands and change support occurs. The matching hypothesis (Cohen and Wills, 1985) predicts that not every act of support eases any kind of strain, but support has to be similar in content to the demand to either buffer the detrimental effect of the demand or develop a direct positive effect (e.g., instrumental support match only with instrumental demands). According to the optimal support matching model (Cutrona, 1990), controllable instrumental demands must also be matched with instrumental support and uncontrollable emotional demands must be matched with emotional support. The triple match principle (de Jonge and Dormann, 2006) of the Demand-Induced Strain Compensation model (DISC; de Jonge et al., 2008), a more recent job stress theory that assumes health restrictions fostered by job demands can best be reduced by matching resources, even goes one step further. The principle states that not only must demands and support dimensionally match, but the interaction is threefold between demands, support, and outcomes (i.e., an affective demand such as strain is best buffered by an affective resource such as emotional support when the outcome is also affective in content such as well-being). Thus, it remains to be tested whether matching the content of the change support to the specific change demand reduces the effect of the change demand more strongly than without matching.

Empirical examination shows mixed results on the assumptions of the interaction between demands and support made by JD-R, matching hypothesis and the triple match-principle. Assumptions of JD-R that job demands and job resources interact to affect job outcomes have been supported empirically by Bakker et al. (2010). Viswesvaran et al. (1999) found meta-analytical support for the buffering effect of social support on the demand-outcome relation with a small but significant moderator effect. Still, the authors also found support for an individual direct effect of demands and support on outcomes. The evidence for the triple match principle is mixed as well. Research (Chrisopoulos et al., 2010; van de Ven et al., 2014; Balk et al., 2020) has shown that the likelihood of finding an interaction effect for different outcomes increases significantly when not just a dual match (as in matching hypothesis) but also a triple match occurs. Yet, the researchers also observed many cases in which no interaction or only a dual interaction occurred. Therefore, the specific conditions under which an interaction can occur, as also stated by Jolly et al. (2021), requires further investigation.

An interaction in accordance with matching hypothesis or triple match principle is more likely to occur when change support is designed to counteract specific change demands, therefore affecting specific change-related outcomes. When technology implementation produces a high amplitude of demands, support might also have to take multiple forms to counteract each demand. When demands are rather instrumental (i.e., new skill requirements after alteration of work tasks), the support intervention also needs to be instrumental in nature (i.e., training option for new work tasks). This should then be most effective for behavioral outcomes such as behavioral intention to use. When the demand is rather emotional in nature (i.e., uncertainty due to altered work roles and collaboration), the support intervention also has to be emotional in nature (i.e., participation option to regain some control of the change process and design one’s new work role). This should most effectively influence affective outcomes such as process satisfaction. Concerning the interaction of change demands and change support, we propose that high change support can reduce the negative effect of high change demands on outcomes, especially when the match is not just dual between demand and support, but also triple with its outcome, as the results of the same dimension should be more affected. When change demands are low and matching change support is also low, no overly negative or positive effects on outcomes are expected, but neutral ones. When change demands are low, but still high change support is presented, we expect that change support will still be viewed positively by employees as it signals interest of the organization, as long as the support is not perceived as forced upon the employee and intrusive (Deelstra et al., 2003; Gray et al., 2020). In either case, we assume that high change support can buffer the negative effect of high change demands on outcomes. We only test for matching of change demand and change support with a parsimonious design (as opposed to a test of mismatch) because we expect matching to lead to larger buffer effects than providing mixed support for mixed demands, as tested in Study 1.

*Hypothesis 4:* An interaction is more likely to occur when change demands, change support, and outcomes match.

## Materials and Methods of Study 2 and Study 3

### Study 2

In Study 2, we analyzed whether a specific instrumental change demand (skill loss after technology implementation) can be buffered by the provision of specific instrumental change support (provision of training).

#### Research Sample

For the second and third studies, we calculated the required sample size with G*Power (Faul et al., 2007) from the estimates of the first study. The field study and the first vignette study resulted in comparable estimates. The *a priori F*-test (ANOVA, main effects and interactions) results showed that between *N* = 63 (attitude to change, *r*_mean_ = 0.34), *N* = 63 (process satisfaction, *r*_mean_ = 0.34) and *N* = 467 (behavioral use intention, *r*_mean_ = 0.13) participants needed to be recruited. Because Maxwell et al. (2015) noted that larger samples sizes are required for replication studies, we increased the sample size accordingly.

For Study 2, participants were recruited in 2020 via the crowdsourcing platform Prolific, and paid 3.50 € for an estimated participation time of 20 min. Participants had to speak fluent German and be resident in Germany. Overall, populations recruited via Prolific show a high response quality (Peer et al., 2017) and a similarity in demographics and responses to conservatively recruited study pools (Behrend et al., 2011; Walter et al., 2019). Several steps to increase sample quality were undertaken. First, Prolific allows for a prescreening of study participants to only include participants that match the intended sample (e.g., working at least 20 h per week) (Palan and Schitter, 2018). Second, several attention checks were administered to check for inattentive participants or fraudulent behavior (Newman et al., 2021), which almost all participants answered correctly. In total, *N* = 154 were recruited for Study 2. After applying the same exclusion criteria as reported for the first study, *N* = 134 were included in the final sample.

Participants were on average 30.31 years old (*SD* = 7.9; range: 18–61 years), 61.9% male. On average, they worked 37.6 h per week (*SD* = 6.32; range: 20–60 h), in a dependently employed (88.1%), marginally employed (3.0%), or part-time employment position alongside studies or training (9.0%). The majority of participants had a university degree (56.7%) or vocational training (17.1%). Most participants held no leadership responsibilities (61.2%), while 36.6% had leadership responsibilities for teams or projects, and 2.2% on some higher hierarchy level. Most participants worked in the natural sciences or informatics (20.9%), health services (20.1%), or commercial services (17.9%). Additionally, 84.3% of the participants had experienced technology implementation in their workplace; on average, they judged the implementation as slightly good (3.73, *SD* = 0.86, range 2–5). Participants evaluated themselves as tech-savvy (4.19, *SD* = 0.91, range 1–5).

#### Procedure and Manipulation

Each of the three studies followed a similar study design. In Study 2, the vignette description was set in the same organizational context. For the manipulation of change demand, we described how specific tasks would change so that study participants would no longer have (high demand) or still have (low demand) the necessary skills required to complete the job. For the manipulation of change support, we described that the employees could take part (high support) or could not take part due to budget constraints (low support) in a training program that would teach them the new skills required for their job.

#### Measures

Each of the three studies assessed the same set of items to ensure the comparability of the study results (Shrout and Rodgers, 2018). Please refer to the description of Study 1.

### Study 3

In Study 3, we analyzed whether a specific emotional change demand (work role changes after technology implementation) can be buffered by the provision of a specific emotional change support (provision of participation options).

#### Research Sample

We applied the same recruitment strategy as in Study 2. In total, *N* = 146 participants were recruited. After applying the same exclusion criteria as reported for the first study, *N* = 138 participants were included in the final sample.

Participants were on average 30.6 years old (*SD* = 8.09; range: 18–64 years), 70.3% male. On average, they worked 37.12 h per week (*SD* = 7.86; range: 20–63 h), in a dependently employed (92%), marginally employed (0.7%), or part-time employment position alongside studies or training (7.2%). The majority of participants had a university degree (63.8%) or vocational training (11.6%). Most participants held no leadership responsibilities (60.1%), while 35.5% had leadership responsibilities for teams or projects, and 4.3% on some higher hierarchy level. Most participants worked in the natural sciences or informatics (31.2%), commercial services (18.1%), or administrative services (14.5%). Additionally, 90.6% of the participants had experienced technology implementation in their workplace; on average, they judged the implementation as slightly good (3.76, *SD* = 0.89; range 2–5). Participants evaluated themselves as tech-savvy (4.34, *SD* = 0.77, range 2–5).

#### Procedure and Manipulation

In Study 3, we applied a similar manipulation as in Studies 1 and 2. For the manipulation of change demand, we described how specific work routines change following the technology implementation and alter the job role the participant holds in the fictitious organization strongly (high demand) or slightly (low demand). For the manipulation of change support, we described that the employees could participate (high support) or could not participate (low support) in the redesign of their job roles and formulate ideas and concerns.

#### Measures

We applied the same set of measures as used in Studies 1 and 2.

## Results and Discussion of Study 2 and Study 3

### Manipulation Check and Descriptive Statistics

The manipulation of change demands was successful [Study 2: *t*(132) = 15.72, *p* < 0.001, *d* = 0.73; Study 3: *t*(136) = 15.77, *p* < 0.001, *d* = 0.74]. The manipulation of change support was also successful. The mean difference for availability of support [Study 2: *t*(132) = 6.57, *p* < 0.001, *d* = 1.13; Study 3: *t*(136) = 13.23, *p* < 0.001, *d* = 0.97] as well as perceived organizational support was significant [Study 2: *t*(132) = 5.87, *p* < 0.001, *d* = 1.28; Study 3: *t*(136) = 17.14, *p* < 0.001, *d* = 1.01].

Mean differences, standard deviations, and correlations of study variables are portrayed in Tables 1, 5. Overall, estimates show in the intended direction.

**TABLE 5:** Correlation matrix of Study 2 and Study 3.

	1	2	3	4	5	6	7
(1) Demand (0 = low, 1 = high)	0.85/0.89	0.01	–0.24**	0.51**	–0.30**	–0.21*	0.00
(2) Support (0 = low, 1 = high)	0.03	0.83/0.91	0.28**	–0.22**	0.33**	0.51**	0.30**
(3) Need satisfaction	–0.39**	0.20*	0.89/0.86	–0.47**	0.54**	0.55**	0.41**
(4) Frustration	0.61^**^	–0.10	–0.59**	0.86/0.76	–0.62**	–0.52**	–0.32**
(5) Attitude to change	–0.50**	0.25**	0.64**	–0.70**	0.93/0.91	0.73**	0.57**
(6) Process satisfaction	–0.41**	0.34**	0.65**	–0.63**	0.79**	0.86/0.81	0.56**
(7) Behavioral use intention	–0.24**	0.17*	0.49**	–0.39**	0.69**	0.61**	0.81/0.81

*Correlations of Study 2 (N = 134) are presented below the diagonal, correlations of Study 3 (N = 138) are presented above the diagonal. Reliability estimates in Cronbach’s alpha on the diagonal.*

*p < 0.05, **p < 0.01.

### Interaction of Change Demands and Change Supports

The results of the analyses of Hypotheses 1 and 2 are portrayed in Table 3. For Study 2, a specific instrumental change demand was manipulated by a description that the technology implementation would lead to a change in task and skill requirements. The results show that the specific instrumental change demand significantly decreased attitude to change (*b* = –1.61, *p* < 0.001), process satisfaction (*b* = –1.85, *p* < 0.001), and behavioral intention to use (*b* = –0.52, *p* = 0.00). The specific instrumental change support, described as the provision of training to compensate skill loss, significantly moderated the relation of change demands and attitude to change (*b* = 0.86, *p* = 0.01) and process satisfaction (*b* = 1.14, *p* = 0.01), but not behavioral intention to use (*b* = 0.30, *p* = 0.24). Therefore, Hypothesis 1 can be supported for all three outcomes, and Hypothesis 2 can be supported for attitude to change and process satisfaction. Hypothesis 4, assuming a stronger relation between matching demand, support, and outcome, cannot be confirmed because there was no interaction with the behavioral outcome behavioral intention to use. However, a dual match between demand and support was confirmed.

For Study 3, a specific emotional change demand was manipulated (a job role change triggered by the technology implementation). The results show that the specific emotional change demand significantly decreased attitude to change (*b* = –0.50, *p* = 0.03), but not process satisfaction (*b* = –0.51, *p* = 0.07) and behavioral intention to use (*b* = 0.23, *p* = 0.17). The specific emotional change support, described as the provision of participation options, did not moderate the relation of change demand and outcomes. However, this change support had a significant direct relation with attitude to change (*b* = 0.82, *p* = 0.00), process satisfaction (*b* = 1.51, *p* < 0.001), and behavioral intention to use (*b* = 0.66, *p* < 0.001). Therefore, Hypothesis 1 can be confirmed for attitude to change, whereas Hypotheses 2 and 4 cannot be confirmed.

### Mechanisms of the Effect of Change Demands

In Study 2 (Table 6), change demands significantly negatively affected need satisfaction (*b* = –0.67, *p* < 0.001), and need satisfaction significantly negatively affected frustration (*b* = –1.10, *p* < 0.001). Frustration, in turn, significantly negatively affected attitude to change (*b* = –0.30, *p* < 0.001), process satisfaction (*b* = –0.33, *p* < 0.001), but not behavioral use intention (*b* = –0.09, *p* = 0.10). Change support significantly moderated the relation of change demands with need satisfaction (*b* = 0.40, *p* = 0.03), but was not directly significantly related to any of the outcomes. The direct effect was not significant for any of the outcomes. Yet, the indirect effect of mediation via need satisfaction, frustration, or both was significant for attitude to change and process satisfaction; mediation via only need satisfaction was significant for behavioral use intention. Hypothesis 3 can be supported for Study 2. Change support moderate the relationship of change demands with outcomes through the mediation of need satisfaction and frustration.

**TABLE 6:** Regression coefficients, standard errors, and model summary information of the moderated mediation model – Study 2.

	Consequent														
	(M1) Need satisfaction			(M2) Frustration			(Y1) Attitude to change			(Y2) Process satisfaction			(Y3) Behavioral use intention		
Antecedent	*b (SE)*	*t*	*p*	*b (SE)*	*t*	*p*	*b (SE)*	*t*	*p*	*b (SE)*	*t*	*p*	*b (SE)*	*t*	*p*
Constant	3.56 (0.09)	38.79	<0.001	6.74 (0.70)	9.73	<0.001	4.36 (0.60)	7.24	<0.001	2.74 (0.83)	3.30	0.00	3.12 (0.55)	5.73	<0.001
(X) Demand	–0.67 (0.13)	–5.11	<0.001	1.49 (0.31)	4.88	<0.001	–0.55 (0.22)	–2.52	0.01	–0.51 (0.30)	–1.69	0.09	–0.06 (0.20)	–0.32	0.75
(W) Support	0.05 (0.13)	0.39	0.70	–0.08 (0.28)	–0.28	0.79	0.10 (0.18)	0.54	0.59	0.41 (0.25)	1.61	0.11	0.09 (0.17)	0.54	0.59
Demand x Support	0.40 (0.18)	2.21	0.03	–0.07 (0.40)	–0.17	0.86	0.47 (0.26)	1.80	0.07	0.60 (0.36)	1.66	0.10	0.09 (0.24)	0.39	0.70
(M1) Need Satisfaction	–	–	–	–1.10 (0.19)	–5.90	<0.001	0.58 (0.14)	4.14	<0.001	0.92 (0.19)	4.80	<0.001	0.39 (0.13)	3.12	0.00
(M2) Frustration	–	–	–	–	–	–	–0.30 (0.06)	–5.23	<0.001	–0.33 (0.08)	–4.07	<0.001	–0.09 (0.05)	–1.68	0.10
	*R*^2^ = 0.22			*R*^2^ = 0.52			*R*^2^ = 0.61			*R*^2^ = 0.57			*R*^2^ = 0.23		
	*F*(3,130) = 12.48, *p* < 0.001			*F*(4,129) = 35.37, *p* < 0.001			*F*(5,128) = 39.28, *p* < 0.001			*F*(5,128) = 33.80, *p* < 0.001			*F*(5,128) = 7.79, *p* < 0.001		
Direct effect (95% CI)	W = 0						[–1.00; –0.12]			[–1.11; 0.09]			[–0.46; 0.33]		
	W = 1						[–0.48; 0.31]			[–0.46; 0.63]			[–0.33; 0.39]		
Ind. effect M1 (95% CI)	W = 0						[–0.72; –0.14]			[–1.03; –0.25]			[–0.56; –0.04]		
	W = 1						[–0.30; –0.03]			[–0.48; –0.05]			[–0.25; –0.01]		
Ind. effect M2 (95% CI)	W = 0						[–0.75; –0.23]			[–0.93; –0.18]			[–0.32; 0.01]		
	W = 1						[–0.75; –0.19]			[–0.93; –0.16]			[–0.32; 0.01]		
Ind. effect M1 + M2 (95% CI)	W = 0						[–0.38; –0.10]			[–0.44; –0.09]			[–0.14; 0.01]		
	W = 1						[–0.21; –0.01]			[–0.24; –0.02]			[–0.08; 0.00]		

*N = 134. Manipulation: work task changes as demand, training as support.*

In Study 3 (Table 7), change demands did not significantly negatively affect need satisfaction (*b* = –0.14, *p* = 0.23), yet need satisfaction affected frustration significantly (*b* = –0.83, *p* < 0.001) and frustration significantly affected attitude to change (*b* = –0.36, *p* < 0.001), process satisfaction (*b* = –0.32, *p* < 0.001), and behavioral use intention (*b* = –0.14, *p* = 0.01). Change support did not moderate any of the relations, yet was significantly positively related with need satisfaction (*b* = 0.42, *p* < 0.001), attitude to change (*b* = 0.38, *p* = 0.047), process satisfaction (*b* = 0.98, *p* < 0.001), and behavioral use intention (*b* = 0.42, *p* = 0.01). The direct effect was not significant for any of the outcomes. The indirect effect via need satisfaction, frustration, and both was significant for all of the outcomes. Therefore, Hypothesis 3 cannot be supported for Study 3. In accordance with Study 1, but in contrast to Study 2, change support did not moderate the relation of change demands with outcomes, yet developed an independent significant effect. As in Study 2, the relation was mediated through need satisfaction and frustration.

**TABLE 7:** Regression coefficients, standard errors, and model summary information of the moderated mediation model – Study 3.

	Consequent														
	(M1) Need satisfaction			(M2) Frustration			(Y1) Attitude to change			(Y2) Process satisfaction			(Y3) Behavioral use intention		
Antecedent	*b (SE)*	*t*	*p*	*b (SE)*	*t*	*p*	*b (SE)*	*t*	*p*	*b (SE)*	*t*	*p*	*b (SE)*	*t*	*p*
Constant	3.28 (0.08)	39.50	<0.001	5.98 (0.62)	9.70	<0.001	4.24 (0.60)	7.13	<0.001	2.56 (0.79)	3.25	0.00	3.03 (0.49)	6.14	<0.001
(X) Demand	–0.14 (0.12)	–1.20	0.23	1.26 (0.25)	5.09	<0.001	0.07 (0.20)	0.37	0.71	0.04 (0.27)	0.16	0.87	0.48 (0.17)	2.90	0.00
(W) Support	0.42 (0.12)	3.52	<0.001	–0.23 (0.26)	–0.91	0.37	0.38 (0.19)	2.00	0.047	0.98 (0.25)	3.87	<0.001	0.42 (0.16)	2.65	0.01
Demand x Support	–0.25 (0.17)	–1.41	0.16	–0.24 (0.35)	–0.69	0.49	–0.14 (0.26)	–0.54	0.59	0.01 (0.34)	0.04	0.97	–0.38 (0.22)	–1.76	0.08
(M1) Need Satisfaction	–	–	–	–0.83 (0.18)	–4.61	<0.001	0.55 (0.14)	3.81	<0.001	0.83 (0.19)	4.37	<0.001	0.38 (0.12)	3.23	0.00
(M2) Frustration	–	–	–	–	–	–	–0.36 (0.06)	–5.66	<0.001	–0.32 (0.09)	–3.74	<0.001	–0.14 (0.05)	–2.68	0.01
	*R*^2^ = 0.15			*R*^2^ = 0.41			*R*^2^ = 0.49			*R*^2^ = 0.50			*R*^2^ = 0.27		
	*F*(3,134) = 8.03, *p* < 0.001			*F*(4,133) = 23.25, *p* < 0.001			*F*(5,132) = 25.15, *p* < 0.001			*F*(5,132) = 26.74, *p* < 0.001			*F*(5,132) = 9.76, *p* < 0.001		
Direct effect (95% CI)	W = 0						[–0.32; 0.47]			[–0.48; 0.57]			[0.15; 0.81]		
	W = 1						[–0.46; 0.33]			[–0.47; 0.58]			[–0.23; 0.43]		
Ind. effect M1 (95% CI)	W = 0						[–0.23; 0.06]			[–0.33; 0.09]			[–0.15; 0.05]		
	W = 1						[–0.37; –0.07]			[–0.55; –0.12]			[–0.27; –0.05]		
Ind. effect M2 (95% CI)	W = 0						[–0.76; –0.23]			[–0.73; –0.14]			[–0.35; –0.05]		
	W = 1						[–0.63; –0.17]			[–0.60; –0.11]			[–0.31; –0.04]		
Ind. effect M1 + M2 (95% CI)	W = 0						[–0.13; 0.03]			[–0.12; 0.03]			[–0.06; 0.01]		
	W = 1						[–0.23; –0.03]			[–0.22; –0.03]			[–0.11; –0.01]		

*N = 138. Manipulation: work role changes as demand, participation as support.*

### Discussion of Study 2 and Study 3

In Study 2 and Study 3, we analyzed whether change demands and change support following principles of the matching hypothesis would lead to an interaction effect and increase in effect sizes. In both studies, change demands decreased technology implementation outcomes. In Study 2, we found an interaction effect for attitude to change and process satisfaction, and in Study 3, we found an interaction effect for behavioral use intention. Therefore, when the match between change demands and change support is increased, an interaction effect is more likely to occur. The relation was mediated through perceived frustration, but results for need satisfaction were inconclusive.

## Meta-Analytic Integration of Study Results

To additionally strengthen the estimation of the true effect, and as another remedy to the replication crisis (Maxwell et al., 2015), we integrated the correlation coefficients of the three studies meta-analytically. We applied random-effects three-level meta-analysis (Cheung, 2015). Across the three studies, change demands affected attitude to change significantly with a large effect (*r* = –0.43, *p* < 0.001, 95% LBCI = [–0.55; –0.29]; Bosco et al., 2015). Although the relation was weaker when change support was provided (high support: *r* = –0.41, *p* < 0.001 vs. low support: *r* = –0.45, *p* < 0.001), the moderation analysis showed a non-significant effect [*x*^2^(*df* = 1) = 0.16, *p* = 0.69]. Change demands significantly affected process satisfaction with a medium sized effect (*r* = –0.34, *p* < 0.001, 95% LBCI = [–0.46; –0.20]). Change support did not moderate the relation [*x*^2^(*df* = 1) = 0.03, *p* = 0.87]. Change demands significantly affected behavioral intention to use with a small effect (*r* = –0.14, *p* < 0.05, 95% LBCI = [–0.30; –0.01]). Again, change support did not moderate the relation [*x*^2^(*df* = 1) = 0.50, *p* = 0.48]. For the meta-analytic integration, we found an overall negative effect of demands, but no interaction effect of change support.

## General Discussion

The aim of the present research was to analyze the effect of change demands and its interrelation with change support on working mechanisms and outcomes in a technological change context, to be able to make recommendations for designing more effective interventions to support change and to provide theoretical clarity on the matching principle of social support theory. We applied the integrative framework of social support theory introduced by Jolly et al. (2021) which relates to JD-R (Bakker and Demerouti, 2007) and SDT (Deci and Ryan, 2000; Deci et al., 2017) to explain the joint effect of social support and demands on change-related outcomes with intrapersonal processes. In an experimental research setting in three consecutive studies and meta-analytic integration of research findings, and as proposed by JD-R model, we found evidence that change demands negatively impact the outcomes positive attitude to change, satisfaction with the change process, and behavioral intention to use the implemented technology, which expands the scarce knowledge about demands. This was true for change demands of varying amplitude and dimension (i.e., instrumental and emotional).

Concerning the interaction with change support, we found two kinds of effects. First, no interaction effect occurred between change demands and change support, but change demands and change support each developed an individual direct effect on outcomes. In these cases (Studies 1 and 3), social support did not compensate for the detrimental effects of change demands, but still added a positive effect because change support in itself provided resources. Second, an interaction effect between change demands and change support occurred (Study 2). In this case, change support did not have an individual direct effect on change outcomes, but fully moderated the relation of change demands and outcomes, as change support provided the resources required to counteract the change demands. This result is representative of the findings of the systematic reviews by Viswesvaran et al. (1999) and Jolly et al. (2021) on social support where in a third of the primary studies a moderating effect of social support was not confirmed, yet a direct positive effect of social support was observed. In accordance with JD-R, both the direct positive effect and interaction effect of change support could be expected. When considering assumptions on dual matches between demands and support, we see that an interaction effect between demand and support occurred in Study 2 where a specific instrumental demand (skill loss) was matched to a specific instrumental support (training). We did not find support for a triple match, either because the interaction with the matching outcomes was not significant (Study 2), or because the interaction of demand and support was not significant to begin with (Studies 1 and 3). The reason that an interaction effect was found in Study 2, but not in Studies 1 and 3, could be that employees are most able to use a matching support resource when the stressful event feels controllable and there is a lack of instrumental resources, as was the case in Study 2 with the skill loss-training scenario. In the case of more diffuse (Study 1 – ample changes) or emotionally demanding and uncontrollable stressful events (Study 3 – role change), non-specific acts of (emotional) support are also valuable to employees. Still, whether a direct positive or interaction effect of change support occurs must result from another determinant.

We propose that, for an interaction to occur, the affected employee has to be aware of the match of demand and resource (dual) or demand, resource, and outcome (triple) and attribute personal relevance to the match. With this awareness, employees will pay higher attention to the match. This match is already more likely to be perceived when demand, resource, and outcome match dimensionally, as assumed in theory (Cohen and Wills, 1985; de Jonge and Dormann, 2006). In practice, an instrumental resource such as technical support from the IT helpdesk might still not be the correct response to the instrumental demand of new skill requirements following work task changes when the behavior of efficient performance with the technology requires the employee to have explicit knowledge and training. The relevance of awareness has already been recognized in other work contexts: Garg et al. (2020) were able to explain the differential effects of Human Resources practices by establishing the construct “HR salience” that was defined as the personal relevance an employee gives an HR practice. HR salience was determined by the characteristics of the HR practice as well as the individual preferences of the employee. Also, psychological distance of construal-level theory (Trope and Liberman, 2010) describes how concrete or abstract an event is perceived. Study 2 represents the highest salience or concreteness of change demands and change support, as the demand for new skill requirements and the training support had a stronger match than the combinations in the other two studies, where mixed demands were matched with mixed support (Study 1) or demands of role changes were matched to a participation option to design the role (Study 3); in order for an interaction to occur here, the employee would have had to have been explicitly told that the support offered was to counteract the specific demand.

We assumed that the effect of change demands on outcomes would be mediated by a lack of satisfaction of psychological needs of SDT that lead to a perception of frustration and in effect decrease positive attitude to change, process satisfaction, and behavioral intention to use, however, the impact of which is mitigated by the provision of change support in accordance with the principles of JD-R, dual and triple match. Mediation analysis of the three studies showed that only for Study 2 was full mediation observed, that is, experiencing the change demand of new competency requirements leads to dissatisfaction of psychological needs, which leads to feeling of frustration, which in turn negatively affects positive change attitude, process satisfaction, and behavioral intention to use. For Studies 1 and 3, the effect of change demands and change support was mediated by a perception of frustration only. Yet, change support had a positive direct effect on need satisfaction in Studies 1 and 3, and significantly moderated the effect of change demands with need satisfaction in Study 2. Therefore, whether the detrimental effect of change demands indeed results from a lack of satisfaction of psychological needs in the workplace is inconclusive. However, we found evidence that the detrimental effect of change demands results from the affective perception of the change process as being frustrating.

### Theoretical Implications and Future Research

To date, little research effort was put into the analysis of change demands as compared to the analysis of change support (Smollan, 2015). We contribute to the field by systematically analyzing the effect of different change demands on technology implementation outcomes and interactions with change support in order to design better change interventions in the future, and to increase our understanding of social support matches theoretically (Jolly et al., 2021). We found that different change demands can have different effects on outcomes. Yet, what is required to further progress the understanding of change demands is qualitative research on which specific change demands can occur during each phase of the different kinds and scopes of organizational change (Street and Gallupe, 2009; Senior and Swailes, 2010). In-depth knowledge of demands would then bring about the opportunity to design change support interventions that specifically match these demands.

Concerning the match of change demands and change support, we contribute to the literature by illuminating the factors that determine whether a match occurs and whether a match of demands and support is of higher practical value than no match. Variables that determine whether a match is perceived should be brought into the research focus to extend beyond the theoretical recommendation that demand, support, and outcome should match dimensionally (Cohen and Wills, 1985; de Jonge and Dormann, 2006). To date, the evaluation of match is made by the researcher, yet the affected employees determine whether they perceive a fit and personal relevance between the demand and support intervention (Garg et al., 2020). Possible research paths are the development of measures that ask respondents whether a match between demand and support was perceived or whether the match had personal relevance to their respective work context, or to design support interventions based on the reports of employees as to which demands they perceive in their workplace.

Concerning the question of whether the effectivity of change support interventions can be enhanced when they match more closely their respective change demand, our results show that whether there is an interaction or not, the efficacy is comparable in magnitude. The explained variance of outcome variables was comparable in the three studies, whether change support had a direct effect or an interaction effect. Yet, intervention research in the field researching a support intervention that was designed to counteract specific change demands should evaluate whether its effect size can be enhanced above the effect sizes observed in this study.

Concerning working mechanisms, we found that the relation of change demands and outcomes was mediated by the affective reaction frustration, but that change demands (not in every scenario) decreased the satisfaction of psychological needs in the workplace. This result could be due to the context of the study, as study participants might not have experienced a decrease of need satisfaction as much as they have experienced in a field study (e.g., Schlicher and Maier, 2019b). Still, our study is among the few that examine dissatisfaction of psychological needs and contribute to the understanding of the role of demands for psychological needs satisfaction. Yet, there might also be other working mechanisms that lead to the development of frustration. Promising explanatory mechanisms lie in the social exchange processes (Cropanzano et al., 2017), which has an effect when employees perceive that an organization does not show them appreciation when change demands are high and change support is not available during a change process and, as a consequence, do not feel obligated to reciprocate in change supportive behaviors.

### Practical Implications

Change demands negatively affect employees during technological change. The stresses of organizational change can affect employee well-being (Day et al., 2017), so it is important that those responsible for change have effective interventions in place, and there is still a great deal of uncertainty about what the best interventions are. Based on our results, whether change practitioners are able to design change support intervention according to the specific change demands (interaction effect), or just provide any kind of support (direct effect), our results show that any form of support provided is useful in affecting technology implementation outcomes positively.

Still, the specific technology implementation in an organization can mean specific demands to employees. What exactly technology implementation means for a specific workplace can best be evaluated through employees as the job holders involved. Therefore, change practitioners are well-advised to start a technology implementation process by conducting organizational diagnosis (McFillen et al., 2013), for example by asking employees in focus groups for their evaluation of the impact of the technology on their jobs. Once the specific change demands of a current situation are identified, change support can be designed with the intention to provide for the specific need. In this way, the support intervention will have higher personal relevance (Garg et al., 2020). In organizations where resources are notoriously limited, the design of change support interventions according to specific change demands might lead to more effective yet also more cost-conscious interventions.

Change practitioners should also look out for the working mechanism of the specific change demand. When they understand why a change process is being perceived as frustrating, a change support intervention can be designed in order to affect this specific working mechanism. In case of SDT, this means that when employees, for example, feel their need for competence threatened, they require training that teaches them the necessary skills.

### Strengths and Limitations

In our study, we applied an experimental vignette study approach in order to be able to systematically manipulate the variables of interest while at the same time controlling for the context of the research (e.g., Aguinis and Bradley, 2014). Experimental vignette studies have been criticized in the past for their lack of generalizability, which affects external validity, recommending for a combination of research strategies to increase validity (Scandura and Williams, 2000). Yet, we decided to stick to the experimental approach in three consecutive studies for two reasons. First, the implications of the replication crisis have led to the conclusion of proving findings by replicating them (Shrout and Rodgers, 2018). In the context of our research question on the interaction of change demands and change support, this can best be conducted experimentally by applying different simulated contexts. Second, there already is one field study that, among other hypotheses, tested the interaction of change support and change demands, but suffered from practical issues solvable by experimental research (Schlicher and Maier, 2019b). As in our Studies 1 and 3, a moderating effect of change support could not be found, but participants also reported only low change demands. Such misbalances in study populations can lead to unclear study results. In experimental research, these misbalances can be compensated for by assigning equal numbers of participants to the experimental groups. Also, it can be assumed that the field study has not found an interaction effect because organizations have not yet designed change support to meet specific change demands, but experimental research can solve this problem. Yet, experiments can not only be conducted as vignettes, but also as laboratory experiments, simulating actual behavior (e.g., Deelstra et al., 2003). We still decided to conduct vignette experiments, because the implementation of a new technology and the demands and support it involves naturally develop over a longer time period that is not amenable to the laboratory setting. In addition, vignette study methodologies ask study participants to provide responses to a hypothetical situation, which compromises external validity if the scenario text does not include contextual information that a real-world scenario would provide. In response to the two aforementioned risks, we focused on recruiting participants that had hands-on work experience as dependently employed personnel in an organization, the vast majority also having experienced technology implementation in the past, in order to fully comprehend the described vignette scenario.

In terms of the specific limitations of the three studies, participants in all three studies had higher educational backgrounds and described themselves as average to highly tech-savvy, which may mean that they find it easier to adapt to technological changes and require less support than less educated or tech-savvy samples. This could affect the generalizability of the results. Study 1 examined a vignette with multiple kinds of demands and support provided, as this most closely resembles a practical scenario in organizations, but at the cost of not being able to attribute the effects to a single demand or support resource. In Studies 2 and 3, the vignette scenario was limited to a single demand and matching support, so only one effect was investigated at a time, but this may have been less realistic to a real-world scenario in which consequences of the technology implementation may be more complex. The “new competency requirement – training” scenario in Study 2 may have been easier for participants to navigate than the “role change – participation in role redesign” scenario in Study 3, which was more abstract. Indeed, Study 3 represented a vignette of emotional demand-support match, where demand might be more related to the job (role change) and therefore have a stronger relationship with outcomes that fall outside the scope of this manuscript, for example commitment. Furthermore, we conducted the manipulation check of change demands using three items from the Job Impact Scale that did not distinguish between different demands or technology implementation impacts, but measured whether the work has changed. Although we believe this measure was sufficient to test whether participants noticed the low-high manipulation of change demands, we also see the need to develop more specific measures to assess change demands or technology impact.

The replication effect for the three studies was not as clear as expected. According to Maxwell et al. (2015), replication studies each provide their own distribution that can result in different effect sizes. For the context of technology implementation, the different nuances of change demands and change support was enough to result in different distributions. Furthermore, the proposed higher personal relevance of the interaction of the change demands skill requirement and the support training was a factor that led to a different mechanism of interaction that should find increased focus in future research.

The effects for the behavioral intention outcome were not as strong as for the other outcomes, which may be because the sample sizes of the three studies were too small. However, the meta-analytic integration across the samples of the three studies also showed no interaction between change demands and change support. More importantly, behavioral intention to use has its theoretical foundation in the technology acceptance model (Davis et al., 1989), where large parts of its variance is explained by a perception of the technology being implemented (perceived ease of use, perceived usefulness). The evaluation of the technology was a factor that was excluded from this study in order to only test the interaction effect of change demands and change support, but in practice this would play a huge role in determining employees’ behavioral intention to use the technology.

## Conclusion

Progressing digitalization and technological changes triggered by COVID-19 lockdowns means for organizations that new technologies need to be implemented in shorter time periods (Cascio and Montealegre, 2016; Dwivedi et al., 2020; Parker and Grote, 2020). Researchers and change practitioners have realized that the implementation can be demanding for employees (Smollan, 2015; Demerouti, 2020) and have designed support interventions (e.g., Iden and Eikebrokk, 2013). Yet, how change demands develop their effect and how change support can be best designed in order to effectively counteract change demands in a cost-saving manner has not been satisfactorily answered. With our research, we provide new evidence on the interaction of change demands and change support and open research fields and design recommendations for practice. We demonstrated that change demands of varying amplitudes and dimensions (i.e., instrumental and emotional demands) negatively affected technology implementation outcomes. An interaction effect between change demands and support that buffered the effects of change demands on outcomes was more likely to occur when demands were specific, instrumental, and controllable, and support matched in content, than when demands were diverse or uncontrollable and emotional. This finding could be explained by a stronger personal relevance attributed by support recipients to these matches between demands and support. The relationship was mediated by perceived frustration, though the results were inconclusive for need satisfaction.

Our research contributes to the literature by describing the causal effects of change demands, highlighting the requirements for establishing a match between change demands and support (i.e., dimensional match and personal relevance), and questioning why change demands have a negative effect in order to develop more effective support measures. Designing effective change management measures is highly relevant in practice. The good news is that support, whether it matches or interacts with change demands, has a positive impact on change-related outcomes through both direct and moderated paths.

## Data Availability Statement

The datasets presented in this study can be found in online repositories. The names of the repository/repositories and accession number(s) can be found below: OSF repository https://osf.io/5x2tk/?view_only=7e1bc26e94f2489f866fc75e3e94b43f.

## Ethics Statement

The studies involving human participants were reviewed and approved by Ethical Committee of Bielefeld University. The patients/participants provided their written informed consent to participate in this study.

## Author Contributions

KS designed the outline of the study, performed the statistical analysis, wrote the draft of the manuscript, and revised the manuscript. KS, JR, and GM contributed ideas to the design of the study. KS and JR collected the data. GM critically reviewed the drafts and supervised this work. All authors contributed to the article and approved the submitted version.

## Conflict of Interest

The authors declare that the research was conducted in the absence of any commercial or financial relationships that could be construed as a potential conflict of interest.

## Publisher’s Note

All claims expressed in this article are solely those of the authors and do not necessarily represent those of their affiliated organizations, or those of the publisher, the editors and the reviewers. Any product that may be evaluated in this article, or claim that may be made by its manufacturer, is not guaranteed or endorsed by the publisher.
