# Supplementary material for: Is a Match Better Than No Match? On the Interaction of Demands and Support During Technological Change
Source: Front Psychol. 2022 Jun 23;13:824010. doi: 10.3389/fpsyg.2022.824010 (PMC9261962; doi:10.3389/fpsyg.2022.824010)
Supplement: Supplementary file 1 [file Table_1.DOCX]

Supplementary Material

**Table 1**

*Overview of manipulation groups*

|  |  |  | Support | |
| --- | --- | --- | --- | --- |
|  |  |  | High | Low |
| Study 1 | Demand | High | Strong work process changes – four kinds of support (information, participation, training, management support) provided | Strong work process changes – four kinds of support (information, participation, training, management support) not provided |
|  |  | Low | Weak work process changes – four kinds of support (information, participation, training, management support) provided | Weak work process changes – four kinds of support (information, participation, training, management support) not provided |
| Study 2 |  | High | Strong task changes and new skill requirements – training provided | Strong task changes and new skill requirements – no training provided |
|  |  | Low | Weak task changes, little new skill requirements – training provided | Weak task changes, little new skill requirements – no training provided |
| Study 3 |  | High | Strong task and work role changes – options to participate provided | Strong task and work role changes – no options to participate provided |
|  |  | low | Weak task and work role changes – options to participate provided | Weak task and work role changes – no options to participate provided |

**Table 2**

*Vignette scenario texts, translated from German*

| **Study 1**  *General introduction*  You work in the accounting department of the construction company ‘Buhrmeister Bauplanung’. Two of your most frequent tasks are the posting of invoices and the preparation of monthly reports. Up to now, these activities at ‘Buhrmeister Bauplanung’ have been performed in word processing programs and then archived on the PC. Currently, a new computer program is being introduced to the company for the execution of these activities.  *High demand, high support*  The processes and procedures of your work will **change greatly** as a result of the introduction of the computer program. The change is that you **will have to carry out all the work steps and monthly reports will have to be adapted to the new computer program**. You must create the data in a format that is compatible with the new computer program, set up the computer program correctly, and check the reports that are automatically generated. The introduction of the new computer program will **bring about a major change in your in your daily work processes**.  For the conversion process, you have **detailed time and schedule and information about the goal** of the how, why and by when the changeover is to be completed. You have **several opportunities** to express wishes and ideas regarding the design of the user interface of the computer program or to participate in the introduction process in other ways. **Training** sessions in which the computer program is presented in detail **are accessible to everyone, timely and tailored to the individual questions of the users**. You will **often encounter understanding on the part of your supervisor** when you have problems with the new computer program and quickly receive appropriate assistance.  *Low demand, low support*  The processes and procedures of your work will **change little** for you due to the introduction of the computer program. The change is that the **computer program will provide you with templates for the preparation of invoices and monthly reports at your disposal**. You can create the data in the same format as before; no further settings in the computer program are necessary. You can create the reports yourself as usual. The introduction of the new computer program does **not bring about any changes in your daily work processes**.  For the changeover process, you **have not received a time or schedule or information about the goal of the implementation**, how, why and by when the changeover should have taken place. You are **not given the opportunity** to express wishes and ideas regarding the design of the user interface of the computer program or otherwise **participate in the implementation process**. For **training** in which the computer program could have been presented in detail and individual problems could have been addressed, **no budget was planned**. Your **supervisor does not have time** to deal with your problems in using the new computer program and you will not receive any further assistance. |
| --- |
| **Study 2**  *General introduction*  You work in the accounting department of the construction company ‘Buhrmeister Bauplanung’. Two of your most frequent tasks are the posting of invoices and the preparation of monthly reports. Up to now, these activities at ‘Buhrmeister Bauplanung’ have been performed in word processing programs and then archived on the PC. Currently, a new computer program is being introduced to the company for the execution of these activities.  *High demand, high support*  The processes and procedures of your work will **change greatly** for you with the introduction of the computer program. The change is that you **will have to adjust all steps in the preparation of invoices and monthly reports to the new computer program**. In the future, you will have to prepare the data in a format compatible with the new computer program, set up the computer program correctly, and check the automatically generated reports. The introduction of the new computer program will thus bring **major changes to your daily work processes**. In concrete terms, this means that you **will not have the necessary skills to perform the new tasks**.  **Training** where the computer program can be presented in detail and individual problems can be addressed is **offered to everyone in an accessible, timely manner and tailored to individual user issues**. There are **training sessions** where you are **taught the required new skills**.  *Low demand, low support*  The processes and procedures of your work will **change little** with the introduction of the computer program. The change is that t**he program provides you with templates for creating invoices and monthly reports**. You can create the data in the same format as before; no further adjustments to the program are necessary. Otherwise, you create the reports yourself as usual. The introduction of the new software thus brings **little change to your daily work processes**. In concrete terms, this means that you will **already have all the necessary skills to handle the new tasks**.  **No budget** has been allocated **for training** sessions in which the computer program could have been presented in detail and in which individual problems could have been addressed. There is **no training in which you are taught the new skills** you need. |
| **Study 3**  *General introduction*  You work in the accounting department of the construction company ‘Buhrmeister Bauplanung’. Two of your most frequent tasks are the posting of invoices and the preparation of monthly reports. Up to now, these activities at ‘Buhrmeister Bauplanung’ have been performed in word processing programs and then archived on the PC. Currently, a new computer program is being introduced to the company for the execution of these activities.  *High demand, high support*  The processes and procedures of your work will **change greatly** for you with the introduction of the computer program. The change is that **you will have to adjust all the steps in the preparation of invoices and monthly reports to the new computer program**. In the future, you will have to prepare the data in a format compatible with the new computer program, set up the computer program correctly, and check the reports that are generated automatically. Thus, the introduction of the new computer program **will bring about a major change in your daily operations**. In concrete terms, this means that your **working routines** for processing tasks **will change greatly**, and **your role in the work process is still unclear**.  You have **multiple opportunities** to contribute wishes and ideas regarding the design of your department’s work or the computer program’s user interface, or to **participate in the implementation process** in other ways. You **can get involved in the design of new work routines and roles yourself**; this will be decided in your department in a timely manner.  *Low demands, low support*  The processes and procedures of your work will change little for you with the introduction of the computer program. The change is that the **program will provide you with templates for creating invoices and monthly reports**. You can create the data in the same format as before; no further adjustments to the program are necessary. Otherwise, you create the reports yourself as usual. The introduction of the new software thus brings **little change to your daily work processes**. In concrete terms, this means that **your work routines for processing the tasks will hardly change and your role in the work process remains as before.**  You will **not have the opportunity** to contribute requests and ideas regarding the design of your department’s work or the computer program’s user interface, or to otherwise **participate in the implementation process.** You **cannot get involved in the** **design of new work routines and roles yourself;** that will be decided elsewhere and later. |
